# Age-Specific Search Strategies for Medline

**DOI:** 10.2196/jmir.8.4.e25

**Published:** 2006-10-25

**Authors:** Monika Kastner, Nancy L Wilczynski, Cindy Walker-Dilks, Kathleen Ann McKibbon, Brian Haynes

**Affiliations:** ^*^for the Hedges Team; ^3^Department of MedicineFaculty of Health SciencesMcMaster UniversityHamiltonONCanada; ^2^Health Information Research UnitDepartment of Clinical Epidemiology and BiostatisticsFaculty of Health SciencesMcMaster UniversityHamiltonONCanada; ^1^Department of Health Policy Management and EvaluationFaculty of MedicineUniversity of TorontoTorontoONCanada

**Keywords:** Information storage and retrieval, Medline, medical subject headings

## Abstract

**Background:**

Many clinicians and researchers are interested in patients of a specific age (childhood, geriatrics, and so on). Searching for age-specific publications in large bibliographic databases such as Medline is problematic because of inconsistencies in indexing, overlapping age categories, and the spread of the relevant literature over many journals. To our knowledge, no empirically tested age-specific search strategies exist for Medline.

**Objective:**

We sought to determine the retrieval characteristics of age-specific terms in Medline for identifying studies relevant for five clinical specialties: adult medicine, geriatric medicine, pediatric medicine, neonatal medicine, and obstetrics.

**Methods:**

We compared age-specific search terms and phrases for the retrieval of citations in Medline with a manual hand search of the literature for 161 core health care journals. Six experienced research assistants who were trained and intensively calibrated read all issues of 161 journals for the publishing year 2000. In addition to classifying all articles for purpose and quality, study participants' ages were also recorded. Outcome measures were sensitivity, specificity, precision, and accuracy of single and combination search terms.

**Results:**

When maximizing sensitivity, the best sensitivity and specificity achieved with combination terms were 98% and 81.2%, respectively, for pediatric medicine, 96.4% and 55.9% for geriatric medicine, 95.3% and 83.6% for neonatal medicine, 94.9% and 64.5% for adult medicine, and 82% and 97.1% for obstetrics. When specificity was maximized, all disciplines had an expected decrease in sensitivity and an increase in precision. Highest values for optimizing best sensitivity and specificity were achieved in neonatal medicine, 92.5% and 92.6%, respectively.

**Conclusion:**

Selected single terms and combinations of MeSH terms and textwords can reliably retrieve age-specific studies cited in Medline.

## Introduction

Clinicians and researchers seeking research reports for specific age categories, including generalists and those who are engaged in clinical specialties such as adult medicine, geriatric medicine, pediatric medicine, neonatal medicine, or obstetrics, need to target their literature searches so that the information they retrieve is relevant to their patient population. Difficulty in finding pertinent evidence contributes to the challenges	health professionals have in keeping up-to-date and practising evidence-based medicine [1-7].

Finding age-specific evidence in Medline is a difficult task for several key reasons. In large bibliographic databases such as Medline, optimal search retrieval for individual topics is hampered by the overwhelming amount of available information that is not pertinent to the question. When users search in Medline they have the potential to retrieve articles from any of the approximately 4800 journals that are currently indexed in the database. The size of this general purpose biomedical database coupled with imperfections in indexing [1-3] lead to a high risk of missing articles that are relevant to the topic of the search while at the same time retrieving many articles that are off target. Effective ways to refine the search may be helpful for those wanting to keep up-to-date and for those looking for an answer to a specific patient care question.

Searching in Medline for a specific patient population by selecting "age-specific" journals will not help because studies relevant to any age group are scattered through a wide range of journals, including general journals that cater to no particular age group. Moreover, in Medline, the indexing practices used to identify the ages of those involved in a study are so liberal that they create a very imprecise representation of the age categories of the participants within the study. Medline indexers apply all relevant age-specific index terms to an article regardless of how many participants fall within that category. Thus, if just one patient or participant in the study falls into a particular age category, that age-specific medical subject heading (MeSH) term will be applied. For example, if a researcher was interested in intercultural communication in family medicine around issues of newborn care, the study by Harmsen and colleagues [8] might be retrieved using the following index terms: infant, newborn; ethnic groups; communication; and family practice. However, looking at the patient population studied, only 0.9% of the participants were children under the age of 12 years—likely very few of these were newborns. The study included participants from many age categories, resulting in eight age-specific index terms being assigned to this article (infant, newborn; infant; child, preschool; child; adolescent; adult; middle aged; and aged). For searchers who are interested in communication around newborn issues, this article is likely not useful even though the indexing indicates that it is potentially relevant. These age-classification problems are compounded by the less than optimal search strategies used by clinicians, including their lack of knowledge about how to narrow searches without missing relevant information, and their uncertainty about when to stop searching [9,10].

To assist clinicians searching for studies on age-specific patient populations, we have developed and tested Medline search strategies for detecting studies for specific age categories as well as tested age-specific search terms pertinent to five age-related clinical specialties. In this paper, we report on the evaluation of the retrieval performance of age-specific search strategies in Medline compared with a manual review (the "gold standard" search) of each article in every issue of 161 journals in the year 2000.

Search strategies are useful tools when searching in large electronic databases. We previously developed search strategies for use in Medline to detect clinically relevant scientifically sound articles in the areas of causation, prognosis, treatment, and diagnosis [11-15]. After publishing our initial work on search strategy development [15], we were approached by neonatologists and gerontologists to develop age-specific search strategies because they expressed frustration with the inefficiency of the current system for finding content specific to their patient population. Using only the age-related MeSH terms when searching can be time-consuming because retrievals can be very large and imprecise. To our knowledge, no empirically developed age-specific search strategies have been previously reported for Medline.

## Methods

The study compared the retrieval performance of age-specific search terms and phrases in Medline (accessed using Ovid) with a manual review of each article in every issue of 161 journal titles for the year 2000. The 161 journals were chosen over several years in an iterative process based on a hand search review of over 400 journals. The journals were recommended by clinicians, librarians, editors, and publishers and were chosen based on Science Citation Index impact factors and ongoing assessment of their yield of studies and reviews of scientific merit and clinical relevance [16] in the production of 4 evidence-based medicine secondary journals (*ACP Journal Club, Evidence-Based Medicine, Evidence-Based Nursing,* and *Evidence-Based Mental Health*). The 161 journals include content for the disciplines of internal medicine (eg, *Annals of Internal Medicine*), general medical practice (eg, *BMJ, JAMA,* and *Lancet*), mental health (eg, *Archives of General Psychiatry, British Journal of Psychiatry*), and general nursing practice (eg, *Nursing Research*).

Six research assistants hand searched the 161 journals for the year 2000 and collected data on age of the study participants according to our hand search categories defined in Table 1. This data collection was part of a larger study in which the research assistants applied methodological criteria to each article in each issue to determine if the article was methodologically sound for seven purpose categories (eg, treatment and diagnosis). All purpose category definitions and corresponding methodological rigor have been outlined in previous papers [4,17]. Research staff were rigorously calibrated for applying all these criteria, including the age classification of study participants, and interrater agreement for application of all criteria exceeded 80% beyond chance κ = 0.81; 95% CI = 0.79-0.84) [4].

**Table 1:** Comparison of hand searching and Medline MeSH classification of age categories

**Hand Search Category**	**Our Definition**	**Medline MeSH Term Category**	**MeSH Definition**
Fetus	Fetus	-	-
Newborn	Birth to 1 month	Infant, newborn	Birth to 1 month
Infant	> 1 month to < 24 months	Infant	1 to 23 months
Preschool	2 years to < 6 years	Child, preschool	2 to 5 years
Child	6 years to < 13 years	Child	6 to 12 years
Adolescent	13 years to < 19 years	Adolescent	13 to 18 years
Adult	19 years to < 45 years	Adult	19 to 44 years
Middle age	45 years to < 65 years	Middle aged	45 to 64 years
Aged	65 years to < 80 years	Aged	65 to 79 years
Aged 80	≥ 80 years	Aged, 80 and over	80 years and over
ND	Nondiscernible	-	-

MeSH terms and textwords related to age (eg, infant, child, adult) were downloaded from Medline and were treated as "diagnostic tests" for detecting studies with an age-specific population as determined by a hand search of the literature from 161 journals (the gold standard). The hand search data were obtained by reading each issue completely. The downloaded Medline data from the 161 journals included the retrieval sets for each of the individual terms. After these two data sources were obtained (ie, the Medline downloads and the hand search review), a database was created that included the matched merged content from these two sources. These Ovid retrieval sets were then manipulated by our own set of programs to calculate our outcome measures—the operating characteristics of each age-specific searching term (eg, sensitivities, specificities, and precision) for individual terms and for combinations of terms. When we merged the two data sets (Medline and hand search), we determined the match. If Medline included an item that was not indexed, we went back to the journal and scored it. If we had scored an item that was not in Medline, we removed it from the merged database. Therefore, the final merged database included only items that had hand search scores and Medline indexing. This merged database was used to develop the age-specific search strategies [17].

Borrowing from the concepts of diagnostic test evaluation and library science, we determined the sensitivity, specificity, precision, and accuracy of single- and multiple-term Medline searches. We considered these operating characteristics as the indicators of search term performance. Sensitivity for a given age-specific topic is defined as the proportion of relevant articles (ie, articles with the desired age-specific content) that are retrieved; specificity is the proportion of nonrelevant articles (ie, articles that are outside the desired age-specific content) not retrieved; precision is the proportion of retrieved articles that are relevant (a library science term that is equivalent to "positive predictive value" in diagnostic test evaluation); and accuracy is the proportion of all articles that are correctly classified (ie, overall proportion of relevant articles retrieved and nonrelevant articles not retrieved). Our hand search of the 161 journals indexed in Medline led to the classification of all articles in these journals for age-related content. Search terms were then tested to determine their performance in retrieving age-relevant articles while eliminating those that were nonrelevant. An automated process (which we developed and implemented using a computer program) was used to calculate the operating characteristics (performance) for each single and combination term in Medline. Formulae for calculating the operating characteristics (ie, sensitivity, specificity, precision, and accuracy) of searches are shown in Table 2.

**Table 2:** Formulae for calculating the sensitivity, specificity, precision, and accuracy of searches for detecting age-specific articles*

	**Hand Search**	
**Detection of Search Terms**	**Meets Criteria**	**Does Not Meet Criteria**
Detected	a	b
Not detected	c	d
	a+c	b+d

^*^Sensitivity = a/(a + c); precision = a/(a + b); specificity = d/(b + d); accuracy = (a + d)/(a +b + c + d). All articles classified during the manual review of the literature, n = (a + b + c + d).

Individual search terms with sensitivity > 25% and specificity > 75% for a given age category were incorporated into the development of search strategies that included two or more terms. All combinations of terms used the Boolean "OR." For the development of multiple-term search strategies to either optimize sensitivity or specificity, we tested all two-term search strategies with sensitivity of at least 75% and specificity at least 50%.

To construct a comprehensive set of search terms, a list of MeSH terms and textwords was initially generated, and input was sought from clinicians and librarians in the United States and Canada through interviews with known searchers, requests at meetings and conferences, and requests to the National Library of Medicine. These experts were asked which terms or phrases they used when searching for age-specific studies, as well when searching for studies in specific purpose categories. Search terms could be MeSH terms, including publication types and subheadings, or textwords specific to age in titles and abstracts of articles. Various truncations were also applied to the textwords, phrases, and MeSH terms. We compiled a list of 543 age-specific terms (Multimedia Appendix). All terms were tested in Medline using the Ovid Technologies searching system.

Age categories for the hand search were modeled from the MeSH terms used to index age content. A comparison of hand search categories and MeSH term definitions is shown in Table 1. The major difference between the hand search age categories and the MeSH terms is in how they were applied. During the hand search, we classified the age of study participants in primary studies or review articles in the following way: select one age category, if possible, or up to three to represent where ≥ 50% of participants fell. This procedure is intended to more accurately represent the focus of age-category research of clinical relevance than the comprehensive indexing of all participants' ages provided by the Medline index terms (which may be more pertinent for nonclinical purposes).

We defined five age-specific specialty areas by collapsing our hand search age categories (see Table 1) and through discussions with clinicians from each specialty area about which definition most appropriately reflected the age of their patients in clinical practice: geriatric medicine (≥ 65 years of age), adult medicine (19 to < 65 years of age), pediatric medicine (> 1 month to < 19 years of age), neonatal medicine (birth to 1 month), and obstetrics (fetus).

## Results

Tables 3 to 7 show the operating characteristics of top-performing combinations of terms with best sensitivity, best specificity, and best optimization of sensitivity and specificity while minimizing the difference between the two, for detecting studies on geriatric medicine, adult medicine, pediatric medicine, neonatal medicine, and obstetrics in Medline in 2000. Search strategies are reported using Ovid's search engine syntax for Medline (mp = multiple posting—term appears in title, abstract, or subject heading; sh = subject heading [MeSH term]; tw = textword—word or phrase appears in title or abstract; : = truncation; pt = publication type; exp = explode—a search term that automatically includes closely related MeSH terms; tu = therapeutic use as a subheading; xs = exploded subheading).

### Geriatric Medicine

The single term "exp adult" yielded the best sensitivity (96.4%) with a specificity of 55.9% for retrieving articles about geriatric medicine. However, by using the next best sensitivity combination, "aged.sh. OR age:.tw.", a small sacrifice in sensitivity (1% absolute decrease) resulted in a much better specificity compared with the most sensitive term (absolute increase 14.4%) and improved precision (absolute increase 5.2%) and accuracy (absolute increase 13.3%). As expected, precision improved slightly when specificity was maximized (absolute increase 8.6%). The term that yielded the best optimization of sensitivity and specificity, "aged.sh.", resulted in 93.6% sensitivity and 82.7% specificity.

**Table 3:** Combination of terms with the best sensitivity, best specificity, and best optimization of sensitivity and specificity for detecting studies about geriatric medicine (≥ 65 years) in Medline in 2000

Search Strategy^*^	**Operating Characteristics^†^**			
	**Sensitivity, %(95% CI)(n = 3309)**	**Specificity, %(95% CI)(n = 45719)**	**Precision, %^‡^(95% CI)**	**Accuracy, %(95% CI)(n = 49028)**
Best sensitivity (exp adult)	96.4 (95.8-97.1)	55.9 (55.5-56.4)	13.7 (13.2-14.1)	58.7 (58.2-59.1)
Next best sensitivity (aged.sh. OR age:.tw.)	95.4 (94.7-96.1)	70.3 (69.8-70.7)	18.9 (18.2-19.4)	72.0 (71.6-72.3)
Best specificity (aged, 80 and over.sh. OR of age.tw.)	63.3 (61.7-65.0)	84.0 (83.7-84.4)	22.3 (21.5-23.1)	82.6 (82.3-83.0)
Next best specificity (aged.sh.)	93.6 (92.8-94.5)	82.7 (82.4-83.1)	28.2 (27.3-29.0)	83.5 (83.1-83.8)
Best optimization of sensitivity and specificity (aged.sh.)	93.6 (92.8-94.5)	82.7 (82.4-83.1)	28.2 (27.3-29.0)	83.5 (83.1-83.8)

^*^Search strategies are reported using Ovid's search engine syntax for Medline (if a single search term is shown, this term outperformed two- and three-term combinations). Best sensitivity while keeping specificity ≥ 50%; Best specificity while keeping sensitivity ≥ 50%; Best Optimization of Sensitivity and Specificity is based on lowest possible absolute difference between sensitivity and specificity; exp = explode, a search term that automatically includes closely related indexing terms; sh = subject heading; : = truncation; tw = textword (word or phrase appears in title or abstract).

^†^Total database has 49028 articles, of which 3309 articles are relevant to geriatric medicine and 45719 are irrelevant to geriatric medicine.

^‡^n varies by row.

### Adult Medicine

The three-term strategy "adult.mp. OR middle aged.sh. OR age:.tw." yielded the best sensitivity (94.9%) and had a specificity of 64.5% for retrieving articles about adult medicine. When specificity was maximized (85.2%) with the single term "middle aged.sh.", sensitivity lowered to 72.3%, but precision improved to 62.1% (absolute increase 14.8%) and accuracy improved as well (absolute increase 9.8%). The best optimization of sensitivity and specificity occurred with the combined terms "middle aged.sh. OR of age.tw.", with values approaching 79%.

**Table 4:** Combination of terms with the best sensitivity, best specificity, and best optimization of sensitivity and specificity for detecting studies about adult medicine (19 to < 65 years) in Medline in 2000

Search Strategy^*^	**Operating Characteristics**^†^			
	**Sensitivity, %(95% CI)(n = 12307)**	**Specificity, %(95% CI)(n = 39721)**	**Precision, %^‡^(95% CI)**	**Accuracy, %(95% CI)(n = 49028)**
Best sensitivity (adult.mp. OR middle aged.sh. OR age:.tw.)	94.9 (94.5-95.3)	64.5 (64.4-64.9)	47.3 (46.6-47.8)	72.1 (71.7-72.5)
Best specificity (middle aged.sh.) Next best specificity (adult.sh.)	72.3 (71.5-73.1) 75.3 (74.6-76.1)	85.2 (84.8-85.5) 81.4 (81.0-81.8)	62.1 (61.3-62.8) 57.6 (56.8-58.4)	81.9 (81.6-82.3) 80.0 (79.5-80.3)
Best optimization of sensitivity and specificity (middle aged.sh. OR of age.tw.)	78.7 (78.0-79.4)	77.9 (77.4-78.3)	54.4 (53.7-55.1)	78.1 (77.7-78.5)

^*^Search strategies are reported using Ovid's search engine syntax for Medline (if a single search term is shown, this term outperformed two- and three-term combinations). Best sensitivity while keeping specificity ≥ 50%; Best specificity while keeping sensitivity ≥ 50%; Best Optimization of Sensitivity and Specificity is based on lowest possible absolute difference between sensitivity and specificity; mp = multiple posting—term appears in title, abstract, or subject heading; sh = subject heading; : = truncation; tw = textword (word or phrase appears in title or abstract).

^†^Total database has 49028 articles, of which 12307 articles are relevant to adult medicine and 39721 are irrelevant to adult medicine.

^‡^n varies by row.

### Pediatric Medicine

The three-term strategy "child:.mp. OR adolescent.mp. OR infan:.mp." yielded the best sensitivity of 98.0% with a specificity of 81.2% for retrieving articles about pediatric medicine. When specificity was maximized (97.1%) with the single term "children.tw.", a striking trade-off in sensitivity occurred as it was lowered to 58.2% (absolute decrease 39.8%). Yet, as expected, precision improved (absolute increase 30.9%). The three-term strategy "adolescent.tw. OR children.tw. OR child, preschool.sh." yielded the best optimization of sensitivity and specificity (89.3% and 87.3%, respectively).

**Table 5:** Combination of terms with the best sensitivity, best specificity, and best optimization of sensitivity and specificity for detecting studies about pediatric medicine (> 1 month to < 19 years) in Medline in 2000

Search Strategy^*^	**Operating Characteristics^†^**			
	**Sensitivity, %(95% CI)(n = 2845)**	**Specificity, %(95% CI)(n = 46183)**	**Precision, %^‡^(95% CI)**	**Accuracy, %(95% CI)(n = 49028)**
Best sensitivity (child:.mp. OR adolescent.mp. OR infan:.mp.)	98.0 (97.4-98.5)	81.2 (81.1-81.4)	24.6 (23.8-25.4)	82.4 (82.1-82.8)
Best specificity (children.tw.)	58.2 (56.4-60.0)	97.1 (97.0-97.3)	55.5 (53.7-57.2)	94.9 (94.7-95.1)
Best optimization of sensitivity and specificity (adolescent.tw. OR children.tw. OR child, preschool.sh.)	89.3 (88.1-90.4)	87.3 (87.0-87.6)	30.3 (29.3-31.3)	87.4 (87.1-87.7)

^*^Search strategies are reported using Ovid's search engine syntax for Medline (if a single search term is shown, this term outperformed two- and three-term combinations). Best sensitivity while keeping specificity ≥ 50%; Best specificity while keeping sensitivity ≥ 50%; Best Optimization of Sensitivity and Specificity is based on lowest possible absolute difference between sensitivity and specificity; mp = multiple posting—term appears in title, abstract, or subject heading; : = truncation; tw = textword (word or phrase appears in title or abstract); sh = subject heading.

^†^Total database has 49028 articles, of which 2845 articles are relevant to pediatric medicine and 46183 are irrelevant to pediatric medicine.

^‡^n varies by row.

### Neonatal Medicine

Best sensitivity (95.3%) was achieved by the three-term strategy "infan:.mp. OR child:.mp. OR gestation:.tw.", with a specificity of 83.6% for retrieving articles about neonatal medicine. An expected trade-off occurred in sensitivity (absolute decrease 41.7%) with the most specific term, "infants.tw." (98.7%). However, precision increased to 38.2% (absolute increase 30.8%) and accuracy reached 98.2%. The three-term strategy "infan:.mp. OR gestation:.tw. OR neonatal.tw." yielded the best optimization of sensitivity and specificity, reaching values of 93% (which were the highest among all five specialties).

**Table 6:** Combination of terms with the best sensitivity, best specificity, and best optimization of sensitivity and specificity for detecting studies about neonatal medicine (birth to 1 month) in Medline in 2000

Search Strategy^*^	**Operating Characteristics^†^**			
	**Sensitivity, %(95% CI)(n = 663)**	**Specificity, %(95% CI)(n = 48365)**	**Precision, %^‡^(95% CI)**	**Accuracy, %(95% CI)(n = 49028)**
Best sensitivity (infan:.mp. OR child:.mp. OR gestation:.tw.)	95.3 (93.7-96.9)	83.6 (83.3-83.9)	7.4 (6.8-7.9)	83.8 (83.4-84.1)
Best specificity (infants.tw.) Next best specificity (infants.tw. OR neonatal.tw.)	53.6 (52.6-60.2) 67.7 (64.0-71.1)	98.7 (98.6-98.8) 98.2 (98.0-98.3)	38.2 (34.9-41.0) 33.7 (31.0-36.0)	98.2 (98.0-98.3) 97.8 (97.6-97.9)
Best optimization of sensitivity and specificity (infan:.mp. OR gestation:.tw. OR neonatal.tw.)	92.5 (90.5-94.5)	92.6 (92.4-92.8)	14.7 (13.6-15.7)	92.6 (92.4-92.8)

^*^Search strategies are reported using Ovid's search engine syntax for Medline (if a single search term is shown, this term outperformed two- and three-term combinations). Best sensitivity while keeping specificity ≥ 50%; Best specificity while keeping sensitivity ≥ 50%; Best Optimization of Sensitivity and Specificity is based on lowest possible absolute difference between sensitivity and specificity; mp = multiple posting—term appears in title, abstract, or subject heading; : = truncation; tw = textword (word or phrase appears in title or abstract).

^†^Total database has 49028 articles, of which 663 articles are relevant to neonatal medicine and 48365 are irrelevant to neonatal medicine.

^‡^n varies by row.

### Obstetrics

The combination of terms "gestation:.tw. OR fetal.tw. OR pregnancy.tw." produced the best sensitivity of 82.0%, with a very high specificity of 97.1% for retrieving articles about obstetrics. The maximization of specificity (reaching almost 99%) with the single term "gestation:.tw." yielded a 1.8% increase in specificity but with a marked trade-off in sensitivity, which decreased to 52.0% (absolute decrease 30%).

**Table 7:** Combination of terms with the best sensitivity, best specificity, and best optimization of sensitivity and specificity for detecting studies about obstetrics (fetus) in Medline in 2000

Search Strategy^*^	**Operating Characteristics^†^**			
	**Sensitivity, %(95% CI)(n = 516)**	**Specificity, %(95% CI)(n = 48512)**	**Precision, %^‡^(95% CI)**	**Accuracy, %(95% CI)(n = 49028)**
Best sensitivity (gestation:.tw. OR fetal.tw. OR pregnancy.tw.)	82.0 (78.7-85.3)	97.1 (97.0-97.3)	23.4 (21.4-25.3)	97.0 (96.9-97.1)
Best specificity (gestation:.tw.)	52.0 (47.6-56.3)	98.9 (98.8-99.0)	33.6 (30.2-36.7)	98.4 (98.3-98.5)
Best optimization of sensitivity and specificity (pregnancy.tw. OR fetal.tw. OR age:.tw.)	80.7 (77.2-84.0)	79.3 (78.9-79.7)	4.0 (3.6-4.4)	79.3 (79.0-79.7)

^*^Search strategies are reported using Ovid's search engine syntax for Medline (if a single search term is shown, this term outperformed two- and three-term combinations). Best sensitivity while keeping specificity ≥ 50%; Best specificity while keeping sensitivity ≥ 50%; Best Optimization of Sensitivity and Specificity is based on lowest possible absolute difference between sensitivity and specificity; : = truncation; tw = textword (word or phrase appears in title or abstract).

^†^Total database has 49028 articles, of which 516 articles are relevant to obstetrics and 48512 are irrelevant to obstetrics.

^‡^n varies by row.

## Discussion

Our study shows that selected age-specific search strategies can achieve high retrieval of studies for age-specific populations. Our age-specific search strategies performed differently among the five specialties we investigated. The highest sensitivity and specificity were achieved for pediatric medicine (98% and 81.2%, respectively) and neonatal medicine (95.3% and 83.6%, respectively). This finding may be a result of these age groups being more precisely defined and that studies tend to be narrowly focused on them. Search strategies within obstetrics yielded a higher specificity (97.1%) than sensitivity (82%), indicating that this strategy was better at filtering out nonrelevant age-specific articles than retrieving them. The best performing strategy for optimizing sensitivity and specificity was achieved within neonatal medicine (92.5% and 92.6%, respectively). In all cases, precision was low, a consequence of searching in large multi-purpose databases. Future research is focusing on potential ways to improve precision without compromising sensitivity, for example, by searching in journal subsets.

A possible limitation to our study is the generalizability of our findings to other publication years as our data was collected in the year 2000. We believe, however, that our search strategies are robust because no major changes have been made to age-specific MeSH terms since the year 2000. Moreover, we have previously shown that search strategies developed in 1990 were robust when searching in 2000 [18]. Another potential limitation of our study is that our interrater agreement for classifying age content did not reach 100%. However, exceeding the level of agreement achieved in our study (> 80% beyond chance) is rarely done in diagnostic studies. The scope of journals investigated in our journal subset could be a limitation, but we have no indication that these search strategies would perform differently in other journal subsets aside from the precision values reported. Precision is affected by the prevalence of on-target articles within the database. Thus, our precision figures are presented as estimates of search strategy performance.

The utility of age-specific filters will vary according to the needs of clinicians and researchers who must weigh the consequences of using a sensitive or specific search. Although a sensitive search will not miss many relevant articles, such searches are less precise and entail time-consuming sorting through irrelevant articles. The narrower yield of a specific search will capture many relevant articles and take less weeding, but it has greater potential for missing key articles.

### Search Examples

To illustrate the use of age-specific search strategies, if a geriatrician was looking for information about current treatment strategies for Huntington disease, she might begin her search by entering the content term "Huntington disease" in Medline, which would yield 5907 articles (Table 8).

**Table 8:** Example: best sensitivity (keeping specificity ≥ 50%) search strategies for detecting treatment studies in geriatric medicine (patients ≥ 65 years of age) in Medline (1996 to July week 3, 2005)

Search Strategy^*^					
**Content Term**	**Boolean Operator**	**Best Sensitivity Combination Strategy for Treatment Studies**	**Boolean Operator**	**Best Sensitivity Combination Strategy for Geriatric Medicine**	**Number of Articles**
Huntington disease	-	-	-	-	5907
Huntington disease	AND	clinical trial.mp. OR clinical trial.pt. OR random:.mp. OR tu.xs.	-	-	901
Huntington disease	AND	clinical trial.mp. OR clinical trial.pt. OR random:.mp. OR tu.xs.	AND	exp adult^†^	483

^*^Search strategies are reported using Ovid's search engine syntax for Medline. mp = multiple posting—term appears in title, abstract, or subject heading; : = truncation; pt = publication type; tu = therapeutic use as a subheading; xs = exploded subheading; exp = explode—a search term that automatically includes closely related indexing terms.

^†^Outperformed two- and three-term combinations.

However, sifting through such a large number of articles would be time-consuming and many of these articles would not be relevant to treatment studies in geriatric medicine. By combining the content term "Huntington disease" with the most sensitive combination of terms for treatment studies (clinical trial.mp. OR clinical trial.pt. OR random:.mp. OR tu.xs.), the search can be narrowed to 901 articles. Further, by adding the most sensitive strategy for geriatric medicine (exp adult) to this search string with the Boolean operator AND, the search is refined to 483 articles, which is much more manageable than the original 5907 articles retrieved from searching the content term only. A sensitive search such as this would be an efficient beginning for researchers interested in conducting systematic reviews.

A more specific approach may be especially useful for physicians who do not have time to process an exhaustive search. In the above example, by combining the content word "Huntington disease" with the most specific search strategy for treatment studies [12], "randomized controlled trial.mp. OR randomized controlled trial.pt.", and the most specific search strategy for geriatric medicine, "aged, 80 and over.sh. OR age.tw.", the search yields five articles (Table 9). This is a dramatic reduction in the number of articles retrieved by searching the content term alone (5907 articles), but key articles can be missed.

**Table 9:** Example: best specificity (keeping sensitivity ≥ 50%) search strategies for detecting treatment studies in geriatric medicine (patients ≥ 65 years of age) in Medline (1996 to July week 3, 2005)

Search Strategy^*^					
**Content Term**	**Boolean Operator**	**Best Specificity Combination Strategy for Treatment Studies**	**Boolean Operator**	**Best Specificity Combination Strategy for Geriatric Medicine**	**Number of Articles**
Huntington disease	-	-	-	-	5907
Huntington disease	AND	Randomized controlled trial.mp. OR randomized controlled trial.pt.	-	-	46
Huntington disease	AND	Randomized controlled trial.mp. OR randomized controlled trial.pt.	AND	aged, 80 and over.sh. OR of age.tw.	5

^*^Search strategies are reported using Ovid's search engine syntax for Medline. mp = multiple posting—term appears in title, abstract, or subject heading; pt = publication type; sh = subject heading; tw = textword (word or phrase appears in title or abstract).

### Conclusion

Selected age-specific search strategies can enhance the retrieval of studies for clinicians and researchers who need information relevant for a well-defined age-category patient population. The optimal trade-off between sensitivity and specificity should be determined according to the needs of the searcher.

## Multimedia Appendix 1

Age terms used for developing age-specific search strategies

## Abbreviations

MeSH: medical subject heading
